# On the Elevated Temperature Thermal Stability of Nanoscale Mn-Ni-Si Precipitates Formed at Lower Temperature in Highly Irradiated Reactor Pressure Vessel Steels

**DOI:** 10.1038/s41598-019-45944-z

**Published:** 2019-07-03

**Authors:** N. Almirall, P. B. Wells, H. Ke, P. Edmondson, D. Morgan, T. Yamamoto, G. R. Odette

**Affiliations:** 1 Materials Department, University of California, Santa Barbara, CA 93106 USA; 2 Department of Materials Science and Engineering Department, University of Wisconsin, Madison, WI 53706 USA; 3 Materials Science and Technology Division, Oak Ridge National Laboratory, Oak Ridge, TN 37831 USA; 4 Present Address: Intel Corporation, Hillsboro, OR 97124 USA; 5 Present Address: Materials Science and Engineering Department, Ohio State University, Columbus, OH 43210 USA

**Keywords:** Metals and alloys, Computational methods, Nuclear energy

## Abstract

Atom probe tomography (APT) and scanning transmission electron microscopy (STEM) techniques were used to probe the long-time thermal stability of nm-scale Mn-Ni-Si precipitates (MNSPs) formed in intermediate and high Ni reactor pressure vessel steels under high fluence neutron irradiation at ≈320 °C. Post irradiation annealing (PIA) at 425 °C for up to 57 weeks was used to determine if the MNSPs are: (a) non-equilibrium solute clusters formed and sustained by radiation induced segregation (RIS); or, (b) equilibrium G or Γ_2_ phases, that precipitate at accelerated rates due to radiation enhanced diffusion (RED). Note the latter is consistent with both thermodynamic models and x-ray diffraction (XRD) measurements. Both the experimental and an independently calibrated cluster dynamics (CD) model results show that the stability of the MNSPs is very sensitive to the alloy Ni and, to a lesser extent, Mn content. Thus, a small fraction of the largest MNSPs in the high Ni steel persist, and begin to coarsen at long times. These results suggest that the MNSPs remain a stable phase, even at 105 °C higher than they formed at, thus are most certainly equilibrium phases at much lower service relevant temperatures of ≈290 °C.

## Introduction

One potential barrier to extending nuclear light water reactor lifetimes to 80 years is embrittlement of their massive reactor pressure vessel (RPV)^1^. Embrittlement is primarily due to the formation of nm-scale precipitates, which cause hardening and a corresponding increase in the ductile-to-brittle transition temperature^2,3^. A major effect of irradiation is radiation enhanced solute diffusion (RED) associated with excess vacancy and self-interstitial concentrations. RED leads to hardening by Cu-rich precipitates (CRP) at low to intermediate neutron fluence (*ϕt*) in steels with more than ≈0.07 at.% Cu^2,4^. Cu is highly insoluble at typical RPV service temperatures (*T*_*i*_) of ≈290 °C and quickly precipitates as coherent bcc clusters that are also enriched in Mn, Ni and Si. Solute-defect cluster complexes, known as stable matrix features, which form in displacement cascades, also cause modest hardening, initially increasing roughly with the square root of *ϕt* in both low Cu and Cu bearing steels^3^. These features are thought to be precursors to formation of well-formed Mn-Ni-Si precipitates at higher *ϕt*, as discussed below.

A main challenge for RPV life extension is predicting embrittlement at low service-flux (*ϕ*) and high *ϕt*, where new embrittlement mechanisms may emerge. Notably, large mole fractions (*f*) of what have been shown to be intermetallic Mn-Ni-Si precipitates (MNSPs), long ago predicted by Odette^5^, form at very high *ϕt* in high *ϕ* test reactor irradiations, both in low Cu and Cu-bearing steels^6^. They have also been observed in much lower *ϕ* test reactor and surveillance irradiations^7–15^. However, MNSPs are not currently treated in US embrittlement models^1^. Odette predicted that MNSPs are enhanced in low-alloy RPV steels by low *ϕ* and *T*_*i*_, high Ni and even trace amounts of Cu^16–18^. The concentration of dissolved Cu in RPV steels range from ≈0.01 to 0.3 wt.%. The corresponding concentration of Mn + Ni + Si is much larger, typically ranging from 2 to 4 wt. %. The latter solutes continue to slowly precipitate long after the matrix Cu is depleted. The MNSPs form as a separate appendage co-precipitate phase on the CRPs in Cu bearing steels, as is observed in thermal ageing studies^19^, low flux power reactor surveillance irradiations^20^, and high flux test reactor irradiations^6^. As noted above, the cascade induced solute-defect cluster complexes, or stable matrix features, are thought to be precursor heterogeneous nucleation sites for MNSPs in low Cu steels, especially at medium Ni contents^21^. Although the existence of MNSPs is not in question, there are a number of unresolved issues regarding their detailed character and formation mechanisms.

Some have argued that MNSPs are not thermodynamic phases, but are rather non-equilibrium solute clusters primarily formed and sustained up to sizes of a few nm by radiation induced segregation (RIS) primarily at dislocation loops that form in displacement cascades^22–25^. Specifically these models suggest either that Mn-Ni clusters are not thermally stable in Fe^22,23^, or that they are only stable at combinations of very low temperatures and high solute concentrations, hence, require RIS to grow and persist^24,25^. In contrast, equilibrium thermodynamic models predict that RED results in large MNSP mole fractions (*f*) at the low RPV operating temperatures of ≈300 °C^26^. Notably, the predicted equilibrium precipitate *f* and compositions are in agreement with atom probe tomography (APT) data from steels irradiated to very high fluence^6^. In addition, recent X-ray diffraction and scattering experiments^27^ have Γ_2_ or G-phase intermetallic crystal structures, consistent with CALPHAD based thermodynamic predictions^21^.

Post irradiation annealing (PIA) can provide significant additional insight into the nature of the MNSPs. For example, clusters that form through a RIS mechanism should not be stable during PIA, even at *T*_*i*_ ≈ 290 °C. However, very slow thermal diffusion kinetics precludes conducting meaningful experiments at such low annealing temperatures (*T*_*a*_). While diffusion rates increase with higher *T*_*a*_, the equilibrium MNSP phase fractions are also reduced. Thus, dissolution of what are argued to be RIS formed Mn-Ni-(Si) clusters following short term anneals at *T*_*a*_ from 450–500 °C^10,28^, or in low solute content model alloys at *T*_*a*_ = 400 °C^14^, does not prove that they are thermodynamically unstable at much lower service *T*_*i*_ ≈ 290 °C. Further, due to their small radii (*r*) of ≈0.50–2.5 nm, even if MNSPs are bulk equilibrium phases, they will dissolve at a higher *T*_*a*_, due to the Gibbs-Thomson effect, if they are below the critical radius in a post-annealing, solute-depleted matrix. The effect of precipitate size is discussed further in Section 4. Note this approach can also be used to estimate the phase boundary at elevated temperature.

Because of the slow diffusion rates below ≈450 °C, very long time (*t*_*a*_) PIA is required to distinguish kinetic from thermodynamic effects, and to explore MNSP phase boundaries for comparison to thermodynamic models, it is absolutely critical to compare the PIA data to predictions of models that properly account for both thermodynamics and dissolution mechanisms and kinetics. Achieving these fundamental objectives also supports refining the predictive Mn-Ni-Si precipitation^21^ and PIA models, including for application to guiding embrittlement predictions and annealing remediation treatments.

## Materials and Methods

The compositions of the two essentially Cu-free split-melt bainitic RPV steels studied here, designated LG and CM6, are shown in Table 1. The split-melt alloy microstructures and properties are fully representative of actual in-service RPV steels. The two steels have similar compositions, with the exception of Ni, that nominally ranges from ≈0.69 at.% (LG, medium) to ≈1.57 at.% (CM6, high). These alloys (among many others) were irradiated in the Advanced Test Reactor (ATR) to a very high fluence, *ϕt* ≈ 1.1 ± 0.2 × 10^21^ n/cm^2^ at a high *ϕ* ≈ 2.3 ± 0.4 × 10^14^ n/cm^2^-s (E > 1 MeV) at ≈320 ± 15 °C^29^. This *ϕt* is ≈11 times higher than that expected for RPVs at extended life, while the corresponding *ϕ* is ≈4600 times higher than typical RPV *ϕ* ≈ 5 × 10^10^ n/cm^2^-s. It is well established that higher *ϕ* delays precipitation to higher *ϕt*, with a *ϕ*-adjusted effective fluence (*ϕt*_*e*_) roughly scaling as *ϕt*_*e*_ ≈ *ϕt*(*ϕ*_*r*_/*ϕ*)^*p*^, where *ϕ*_*r*_ is a specified reference flux and *p* ranges from ≈0.15 to 0.25^2,5,16,30,31^. Thus the effective ATR *ϕt*_*e*_ is estimated to be only ≈2–4 × 10^20^, which is 2 to 4 times the maximum *ϕt*_*e*_ ≈ 1 × 10^20^ that an RPV would be expected to experience at an 80 year extended life.

**Table 1:** Nominal steel compositions (at.%).

Alloy	Cu	Ni	Mn	Mo	P	C	Si	Fe
LG	0.01	0.69	1.36	0.31	0.009	0.73	0.43	bal.
CM6	0.02	1.57	1.50	0.31	0.012	0.68	0.33	bal.

Note, the exact relationship between the effective fluence of the ATR irradiation condition and that experienced under extended life is not critical to the main purpose of this experiment, which was to generate significant quantities of MNSPs that could be readily characterized and modeled under long-time, high-temperature PIA. Atom probe tomography (APT) studies show that in the as-irradiated (AI) condition the alloys are nearly fully decomposed, at an approximately saturated MNSP *f*^6^. The two steels were annealed in vacuum for times of 1, 7, 17, 29 and 57 weeks. The MNSPs were characterized by APT up to 29 weeks, and by Scanning Transmission Electron Microscopy (STEM)-Energy Dispersive X-ray Spectroscopy (EDS) at 57 weeks. The annealing times were selected to ensure that any changes, or lack thereof, in the MNSPs would not be limited by slow solute thermal diffusion kinetics. Due to the very limited amount of irradiated material, the PIA was performed on 1.5 mm punched discs, precluding a sequence of standard microhardness measurements.

### Atom probe tomography

Atom probe tomography (APT) was used to measure the MNSP composition, size distribution and average radius (<*r*>), number density (*N*) and mole fraction (*f*) in the AI condition and following each anneal for all times but the longest annealing time of 57 weeks. The APT was carried out at the Center for Advanced Energy Studies (CAES) located in Idaho Falls, ID, with support from the Idaho National Laboratory managed Nuclear Science User Facilities. APT tips were fabricated using a FEI Quanta 3D FEG Focused Ion Beam, using 5 kV and 2 kV cleanup steps to reduce Ga damage. The tips were examined in a CAMECA LEAP 4000X HR in voltage mode, at a 20% pulse fraction and 50 K. Note one tip of the high Ni steel (CM6), annealed for 29 weeks, was run in laser mode with a pulse energy of 75 pJ, a repetition rate of 250 kHz and a temperature of 40 K, in anticipation that only a very low number density of MNSPs would remain in this condition, so a larger sample volume was required to increase the probability of observing them. However, an MNSP with similar size and composition was also seen in a shorter voltage mode run as well. A full description of the APT analysis procedures can be found in^6^.

APT reconstructions and analysis were performed using the CAMECA Integrated Visualization and Analysis Software (IVAS). The clusters were defined using the cluster analysis tool in the IVAS software with order = 5, *d*_*max*_ = 0.6 nm, *N*_*min*_ = 20–30 and envelope = erosion = 0.6 nm. A constant *d*_*max*_ was used for all conditions. Decreases in d_max_ in a given tip results in a lower measured *f* and <*r>*. Thus, measuring changes in *f* and <*r*> using a different *d*_*max*_ for each annealing interval could introduce artificial biases. The main consequence of choosing a *d*_*max*_ that is too large is that random solute density variations in the matrix might be misidentified as clusters. Note in the AI condition, the solutes are highly depleted from the matrix; hence, the probability of identifying random fluctuations as clusters is negligible. However, significant precipitate dissolution occurs after long-term annealing at 425 °C, resulting in a much higher matrix solute contents. In these cases, all measured precipitates had N» N_min_, so no random solute density fluctuations were incorrectly identified as precipitates. The MNSP *f* was defined as the number of solute atoms in the clusters divided by the total number of atoms in the analyzed volume. The precipitate mole fraction varies slightly from volume fraction if the atomic densities of the precipitate and matrix phases differ. Thus, all model data shown for comparisons is also mole fraction. For further information regarding this difference see the Supplemental Information.

The low evaporation field of the precipitates changes the local magnification factor resulting in a focusing of matrix atoms into the precipitate region on the detector and is signaled by higher than physical atom densities in the reconstructed dataset^32–37^. These artifacts can result in distortions of the composition, shape, and size of precipitates, and most specifically their apparent Fe content, some or all of which actually comes from the adjoining matrix thus contributing to the higher than physical atom density in the precipitate region. Using the number of solute atoms to define the cluster size minimizes these field evaporation distortions. The number of solute atoms associated with each precipitate, corrected for efficiency, was determined and multiplied by the atomic volume of Fe. The precipitate *r* was then defined as the radius of a sphere encompassing the total solute volume. While these precipitates are thought to be intermetallic phases, differences in their corresponding average atomic volume versus Fe results in variations in *r* of less than 2%. The MNSP number density (*N*) was calculated by dividing the number of clusters in the dataset by the total volume in the analyzed tip. Precipitates on the edge of the tip are not included in the determining the size distributions, or average <*r*>, but are counted as one half in the estimating N. The associated error f, N and <*r*> estimates are based on the tip-to-tip variations observed here for conditions with multiple tips, or in the one case with a single tip, the average of the others. The standard IVAS reconstructions, as usual, suggested that there is a significant amount of Fe in all of the MNSPs. While, as noted above, Fe is thought to be an APT artifact, the nominal value is provided for those that wish to interpret the data differently.

### Energy dispersive X-ray spectroscopy

At longer annealing times, a significant reduction in the precipitate number density was observed. While APT has very high spatial resolution and measures the detailed chemical nature of the precipitates, it has a very small sampling volume, making it difficult to characterize them when they are present at a very low number density (<≈10^22^ m^−3^). Thus, at the longest annealing time (57 weeks), Energy Dispersive X-ray Spectroscopy (EDS) was performed using an FEI TALOS F200X S/TEM in the Low Activation Materials Development and Analysis Laboratory at Oak Ridge National Laboratory. EDS mapping was performed using a probe size of ~1 nm and current of 1.0 nA, respectively. Analysis of the data was performed using the Bruker-Esprit software. While the TALOS provided high-resolution chemical maps, it was not fully calibrated for quantitative chemical analysis. Thus to complement these high resolution maps, additional EDS scans were performed on the FEI Titan 300 kV FEG S/TEM at UCSB. A line scan with 4 nm spacing between points was taken across three grains, one with a high density of very large precipitates and two with a few sparse precipitates present, to measure the local solute contents.

### Thermodynamic and cluster dynamics modeling

**Figure 1:**
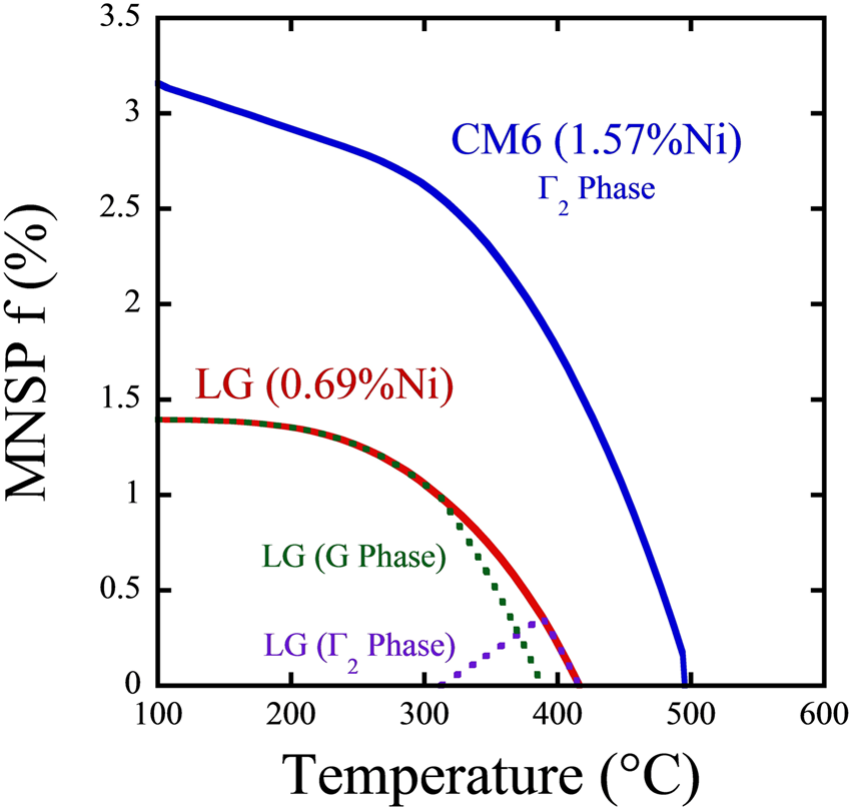
CALPHAD predictions of Mn-Ni-Si precipitate *f* as a function of annealing temperature for two Cu-free steels with varying Ni content.

A cluster dynamics (CD) model, using CALPHAD thermodynamics, thermal diffusion coefficients from literature and fitted precipitate interface energies, was used to guide the experimental design and to help analyze the annealing results [21]. The model predictions of the equilibrium *f* have been reported previously and qualitatively favorably compare to the high *ϕt* ATR data^6^. The corresponding CALPHAD predicted equilibrium *f* for the two low Cu steels as a function of *T*_*a*_ are shown in Fig. 1 for the nominal alloy compositions^26^. Again, the data from all models presented here gives precipitate mole fraction for comparison with APT data. CALPHAD predicts that only the Γ_2_ phase (Mn(Ni,Si)_2_) is stable in the high Ni (CM6) steel, with composition about 33%Mn-52%Ni-15%Si, exists up to 500 °C, while the G phase (Mn_6_Ni_16_Si_7_) persists only up to ≈390 °C in the medium Ni (LG) steel^26^. Note that this bulk phase CALPHAD thermodynamic calculation leading to Fig. 1 does not include effects of the interface energies of the small precipitates, although they are included in the CD model discussed below. Again a recent XRD study for the as-irradiated condition found G phase precipitates in the medium Ni steel, while the high Ni steel contains the Γ_2_ phase, both as predicted by CALPHAD at 320 °C^27^.

Figure 1 shows that CALPHAD predicts that the MNSPs in the medium Ni steel (LG) should completely dissolve above ≈415 °C, while the Γ_2_ phase in high Ni steel (CM6) is predicted to fully dissolve at ≈500 °C. Because lower *T*_*a*_ results in slower solute diffusion, the isothermal annealing was carried out at an intermediate temperature of 425 °C, with the intention of testing the thermodynamic model predicting full dissolution in LG (medium Ni) and that some MNSPs would remain in CM6 (high Ni). The complete MNSP dissolution of the phase in the medium Ni steel also acts as a kinetic marker to help estimate the effective diffusion distances at various annealing times that are pertinent to both alloys.

CD modeling was also carried out to help interpret the very complex MNSP dissolution and coarsening processes. Briefly, CD models the evolution of the MNSPs in discrete n − 1, n and n + 1 cluster sizes, where n is the number of atoms. The n ranges from 2 to n_max_ in a coupled set of n_max_ - 1 ordinary differential equations, which incorporate n-dependent effective solute impingement and emission transition rate coefficients. In this case the solutes are treated as stoichiometric molecules of the pertinent phase. The CD method applied to modeling G and Γ_2_ phase precipitation under irradiation is described elsewhere^21^. The CD model for annealing used here assumes thermal-diffusion controlled kinetics, and requires only 4 key experimental input parameters: (a) the effective thermal solute diffusion coefficient (D), derived from the literature; (b) the solute equilibrium solubility (X_e_), determined by the free energy difference between the dissolved and precipitated solute states (that is the equilibrium phase diagram), evaluated from the Thermo-Calc^38^ TCAL2 database^39^; (c) the MNSP-Fe interface energy (γ), which differ slightly for the G and Γ_2_ phases; and (d) the as-irradiated MNSP size distribution, taken directly from the APT measurements. The γ were derived independently as fit parameters in the CD precipitation model, and were not adjusted in this PIA study^21^. Thus, the PIA CD model has no independently adjusted fit parameters.

## Results and Discussion

### STEM Observations

The APT data on the 29 weeks annealed high Ni alloy showed a very large reduction in the number densities (N) of the MNSPs, with few, if any, precipitates in a given tip. The tips without MNSPs coincided with lower local Ni and Mn contents. Hence, we first focus on the STEM-EDS characterization of the 57 weeks PIA condition, in order to significantly increase the sampling volume relative to the APT observations. This directly confronts the question of the thermodynamic stability of the MNSPs, given a sufficient alloy Ni and Mn content. The STEM-EDS observations generally show regions with no precipitates and other regions with coarsened precipitates still remaining. A typical region with Mn-Ni-Si precipitates, with <*r*> ≈ 2.7 nm versus ≈1.53 nm in the AI condition, is shown in 2a–e. The main conclusion of the STEM-EDS study is that sufficiently coarsened MNSP are stable at near nominal amounts of Ni and Mn, even at a very long *t*_*a*_ that is ≈8 times that required for full dissolution of the MNSPs in the medium Ni alloy.

**Figure 2:**
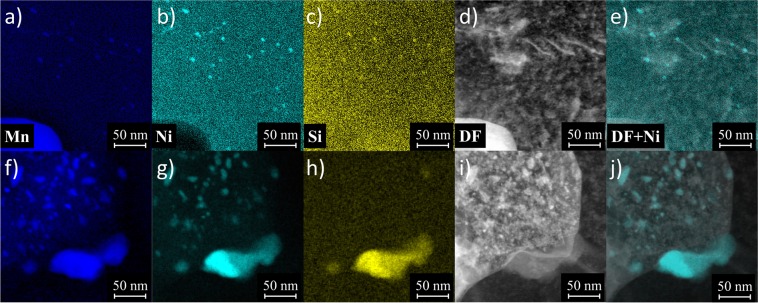
EDS maps showing Mn-Ni-Si precipitates remaining in the high Ni steel after annealing for 57 weeks at 425 °C from: (**a**–**e**) a region with relatively small precipitates, many of which are located on dislocations and (**f**–**j**) a region with very large Mn-Ni precipitates.

Figure 2d is a dark field (DF) STEM image showing a number of dislocation lines. Figure 2e is the same DF image overlaid with a partially transparent image of the Ni EDS signal, clearly showing a strong association between the dislocations and the remaining precipitates. This association is not unexpected, since the energy of Mn-Ni solute clusters are lower on dislocations than in the matrix^24,40^ and, as a corollary, dislocations are attracted to MNSPs.

Figure 2f–j shows one grain that contained very large Mn-Ni enriched features, on the order of 20–30 nm long. These large precipitates were not significantly enriched in Si, except for one even larger MNSP on a grain boundary. The Mn-Ni enriched features are much larger than any previously reported precipitates in a neutron irradiated RPV steel. It is likely that they are a MnNi B_2_ phase^41^. Note, such large features have also been reported following proton irradiation^42^. A FEI Titan STEM EDS line scan was performed to determine the local solute composition of the grain with large precipitates, as well as the two nearby grains which had only a few, sparsely-spaced MNSPs. This scan showed the local composition of the grain with large features was ≈3.36 at.% Ni, 1.23 at.% Mn and 0.34 at.% Si, while both neighboring grains had compositions of ≈1.66–1.70 at.% Ni, 0.60–0.72 at.% Mn and 0.36–0.48 at.% Si. The composition of the very high Ni grain is consistent with the formation of the MnNi B_2_ phase with lower Si^41^. Additional details regarding the line scan can be found in the Supplemental Information.

The most important result from the STEM-EDS study is that, given sufficient Ni, sufficiently large MNSPs are thermodynamically a stable equilibrium phases at 425 °C, which is 105 °C higher than *T*_*i*_, and 135 °C higher than for normal RPV service conditions. Figure 2 shows that the MNSPs are much more stable, and unambiguously thermodynamic equilibrium phases, at these lower *T*_*i*_.

### APT results

**Figure 3:**
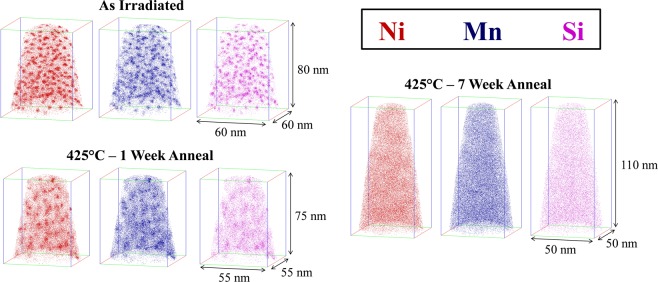
Atom maps for the Cu-free, medium Ni steel (LG) in the (**a**) AI condition, (**b**) 425 °C - 1 week annealed condition, and (**c**) 425 °C annealed - 7 weeks condition.

Atom maps from the medium Ni steel (LG) for the AI and 425 °C PIA conditions are shown in Fig. 3. The MNSPs are very clearly dissolving following the one-week anneal; and the Mn and Si appear to have diffused further than the Ni, hence are the most dilute. The solutes in the medium Ni steel are nearly entirely dissolved after the 7 weeks anneal, as predicted by the thermodynamic model (see Fig. 1), with only weak indications of residual solute clustering. The solutes are expected to be fully dissolved in the medium Ni steel after the 29 weeks anneal, since they have presumably diffused approximately twice as far compared to the 7 weeks condition^43^.

**Figure 4:**
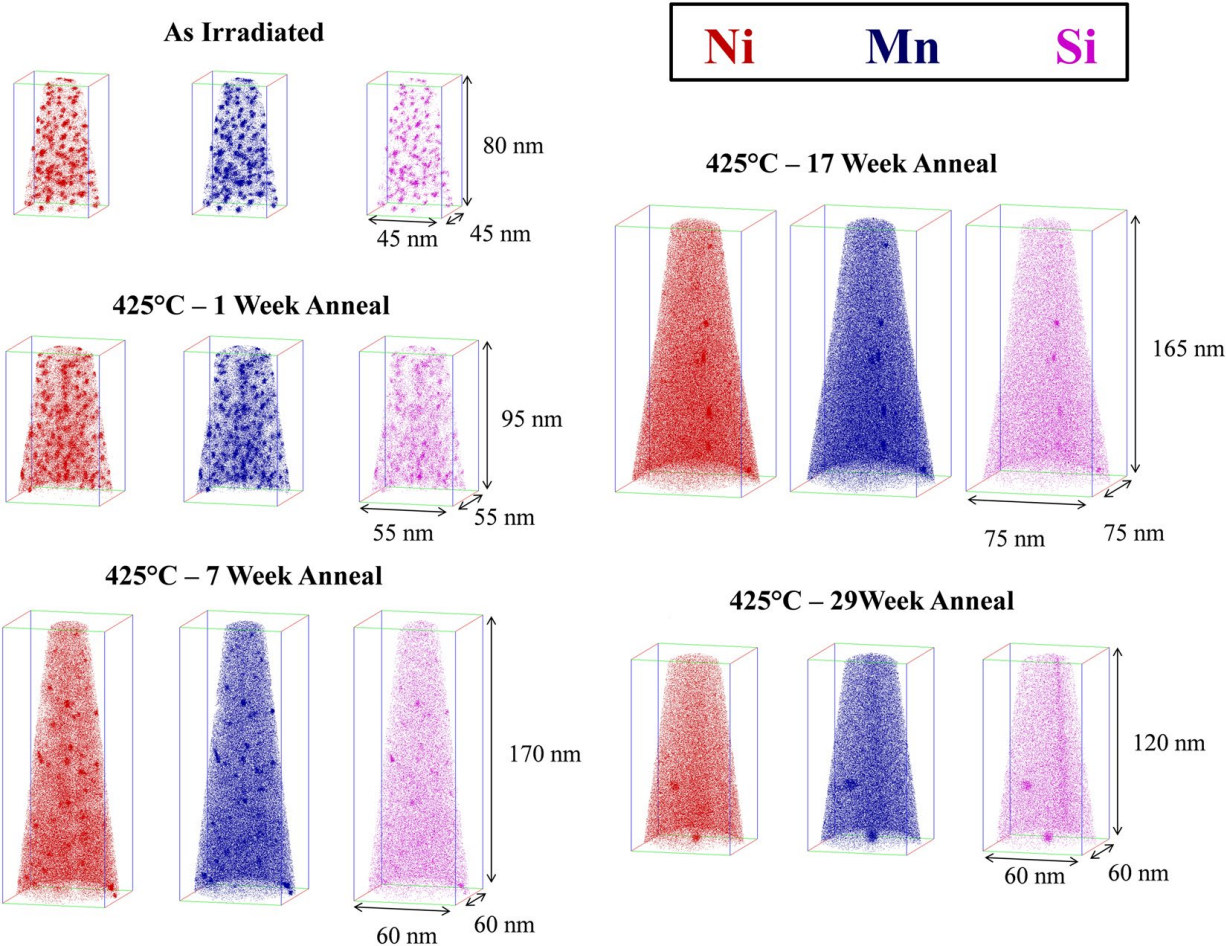
Atom maps for the low Cu, high Ni steel (CM6) in the AI condition (top left) and 425 °C annealed conditions at times of: 1 week (mid left), 7 weeks (bottom left), 17 weeks (top right) and 29 weeks (bottom right).

**Figure 5:**
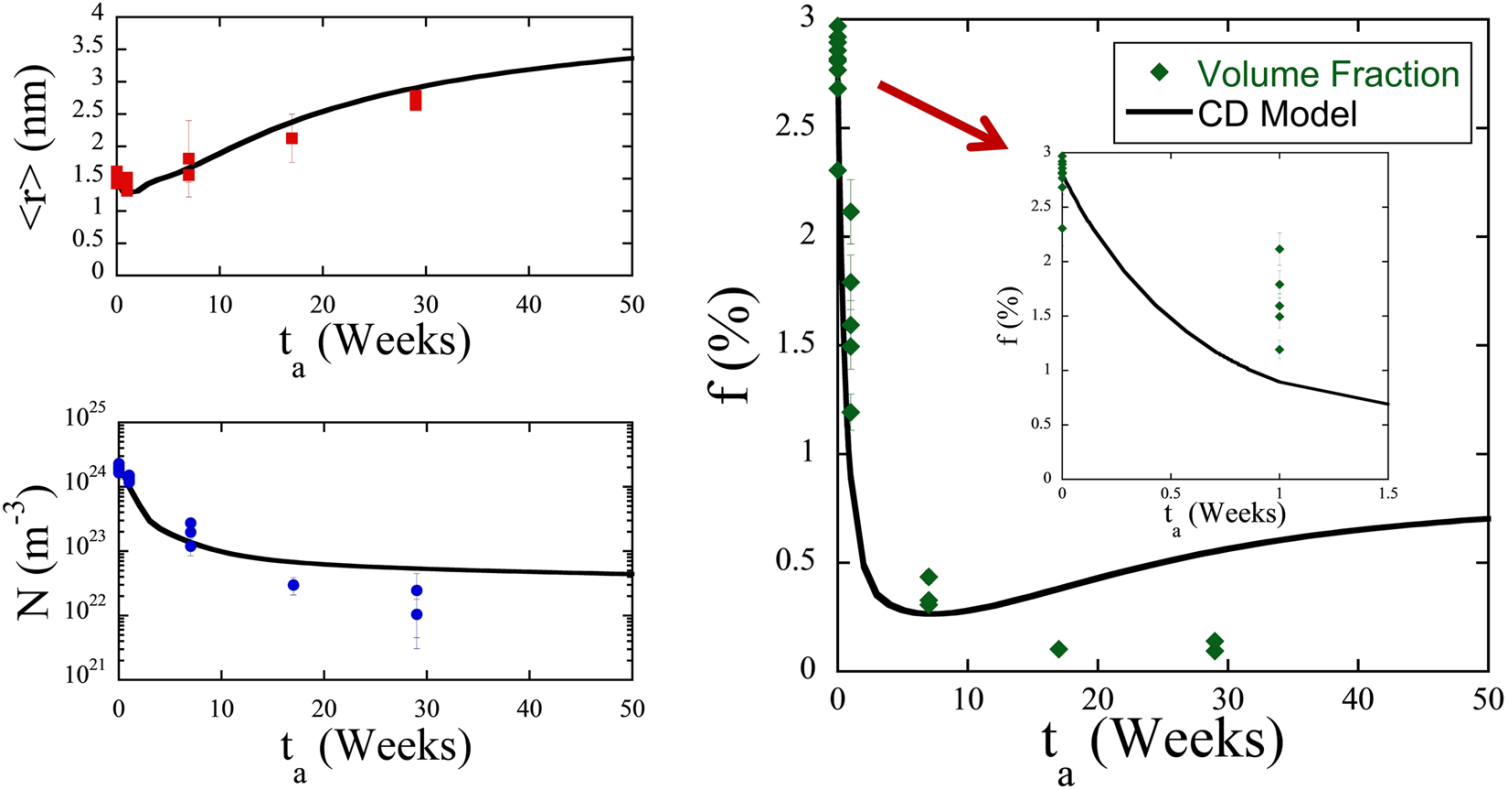
APT measured precipitate <*r*> (nm), *N* (m^−3^) and *f* (%) after annealing (points) and CD predictions (lines) for the high Ni steel (CM6) at *T*_*a*_ = 425 °C. Note that the plot of *f* vs *t*_*a*_ includes a blowup of the shorter annealing times to more clearly see these values. CD simulation conditions are described in the text.

**Figure 6:**
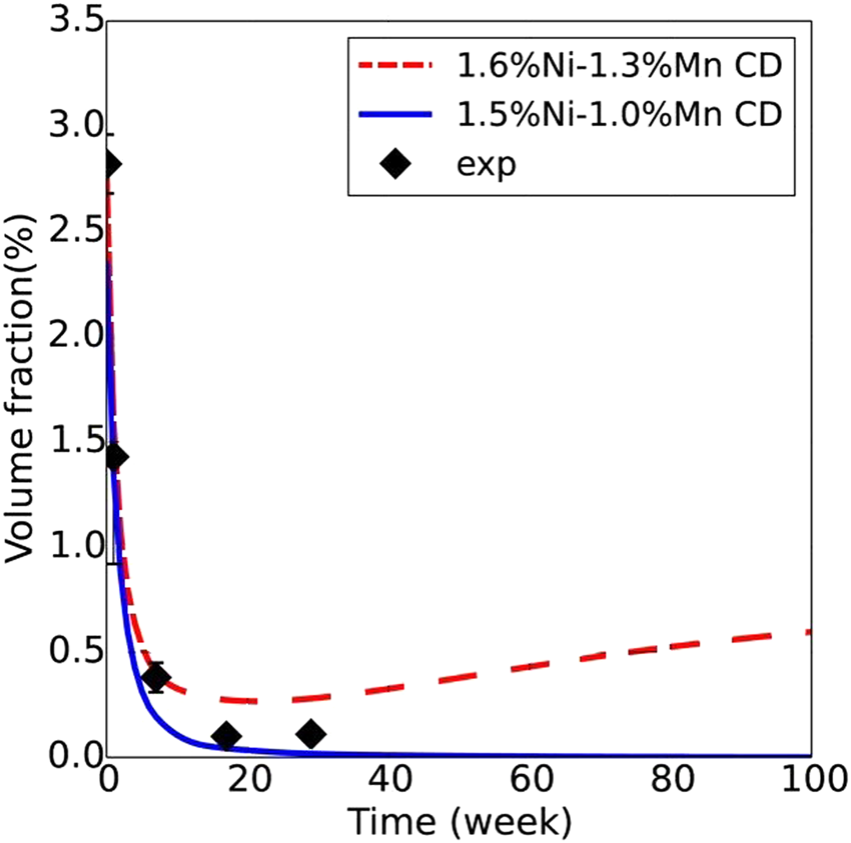
*f* as a function of annealing time at *T*_*a*_ = 425 °C for two CD models with 0.34 at.% Si and either 1.6 at.% Ni and 1.3 at.% Mn or 1.5 at.% Ni and 1.0 at.% Mn.

Atom maps in Fig. 4 for the high 1.6 at.% Ni steel (CM6) show that the MNSPs are much more stable, with well-defined precipitates still remaining after PIA for 29 weeks. The corresponding APT <*r*>, *N* and *f* data are summarized in Table 2 and Fig. 5. Note that Table 2 gives the average values and uncertainty estimates for a given condition, while Fig. 5 shows a data point for each measured APT tip, demonstrating that N and *f* vary significantly from region to region. As will be discussed below, this variability is dictated by the local bulk composition of an individual APT tip. The solid lines in Fig. 5 are the CD model predictions for the nominal composition. Both *N* and *f* decrease rapidly with the increasing *t*_*a*_. There is a corresponding small dip in <*r*>, followed by a slight increase up to 7 weeks, which is primarily due to the dissolution of the smallest MNSPs, rather than significant coarsening of the larger ones. However, it is notable that between 7 and 29 weeks <*r*> closely tracks the kinetics predicted by the CD model. The initial decrease in the MNSP N is also in agreement with model, but the APT data fall below the CD predictions between 17 and 29 weeks, although the rate of decrease in N and f slow, as is expected, under mixed dissolution and coarsening kinetics. Clearly, the nominal CD model over predicts N and f at long times. However, this is not surprising given the approximate parameterization of the CD model and the complexity of the interacting and competing processes mediating precipitate annealing, as discussed below. Note, the continuing decrease in N during PIA would make MNSPs very unlikely to be found in APT studies at 57 weeks, while they are clearly present in the STEM-EDS observations. Figure 6 shows the high sensitivity of the model to modest reductions in alloy Ni and Mn contents, of ≈6 and 23%, respectively, that lead to complete MNSP dissolution, again consistent with the STEM-EDS observations of lower solute regions.

**Table 2:** Precipitate summary for the high Ni steel (CM6) from the AI and 425 °C annealed conditions.

*t*_*a*_	Average *t*_*a*_, <*d*>, *N* and *f*^a^			Average Precipitate Composition (at.%)							Precipitate Fe	Relative Composition
	<*r*>	*N*	*f*	Cu	Ni	Mn	Si	Mo	C	P	Fe^b^	Mn/Ni/Si
0	1.50 ± 0.11	19.50 ± 1.47	2.82 ± 0.14	0.0	52.1	35.2	11.7	0.5	0.5	0.0	58.9	0.35/0.53/0.12
1	1.41 ± 0.19	11.80 ± 2.72	1.43 ± 0.53	0.3	53.0	35.4	10.0	0.8	0.3	0.3	62.1	0.36/0.54/0.10
7	1.63 ± 0.42	2.19 ± 0.70	0.38 ± 0.07	0.0	52.8	38.0	7.8	0.9	0.2	0.2	57.6	0.38/0.54/0.08
17	2.13 ± 0.22	0.30 ± 0.08	0.10 ± 0.02	0.0	52.6	37.6	7.0	1.6	0.9	0.2	55.8	0.39/0.54/0.07
29	2.78 ± 0.13	0.14 ± 0.07	0.11 ± 0.02	0.0	35.8	35.5	14.3	8.7	5.1	0.6	37.5	0.41/0.42/0.17

^*a^Units: *t*_*a*_ (wks), <*d>* (nm), *N* (10^23^ m^−3^), *f* (%). ^b^The nominal IVAS Fe found in all the MNSPs, that is thought to largely be an artifact.

**Figure 7:**
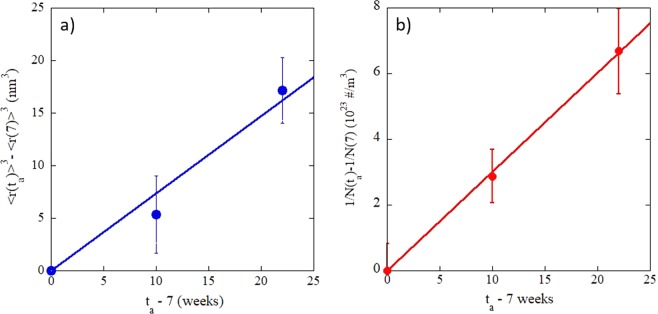
Plots showing MNSP coarsening kinetics that are consistent with a diffusion controlled mechanism for: (**a**) of <r(t_a_)> − <r(7)>^3^; and, (**b**) 1/N(t_a_) − 1/N(7).

The APT observations of MNSP response to PIA at temperatures much higher than they were formed at shows 3 stages: a) initial rapid dissolution of small precipitates between 0 and 7 weeks; b) mixed dissolution and coarsening between 7 and 17 weeks; and, c) coarsening between 17 and 29 weeks at a near minimum f. The kinetic order of coarsening beyond 7 weeks, as reflected in the time exponent, is illustrated in Fig. 7, showing plots of <r(t_a_)>^3^ − <r>(7)>^3^ and 1/N(t_a_) − 1/N(7), which are both approximately linear in t_a_, for classical diffusion controlled coarsening, often called Ostwald Ripening^44^. Notably, the order of coarsening kinetics is insensitive to the various CD model parameters as a combination of interface energy, solubility and diffusion coefficients, which strongly influence the absolute predictions of <*r*>, N and f. As discussed further below, the annealing processes in this case are more complex than those that are treated in simple coarsening models. However, the approximately linear t_a_-dependence of <r(t_a_)>^3^ and 1/N(t_a_) kinetics is powerful evidence that coarsening is occurring during the high temperature PIA at longer t_a_. The <*r*> at 17 weeks is a little lower than the mean fit line, likely due to the transition from dissolution to coarsening dominated kinetics; while 1/N(t_a_) is almost perfectly linear. In both cases the linear kinetics are within the estimated scatter in the data.

The average precipitate compositions as a function of t_a_ are also shown in Table 2. While Fe is an APT artifact, the nominal IVAS value is included in the table for those that wish to interpret the data differently. Table 3 summarizes the corresponding matrix compositions, which return to near nominal bulk values, reflecting the small residual f of the MNSPs. Table 4 shows the relative precipitate Mn, Ni and Si compositions compared to the closest known Mn-Ni-Si intermetallic phases. The Γ_2_ phase (33%Mn-52%Ni-15%Si) is closest to the composition in the AI condition, but the MNSPs contain less Si for t_a_ up to 17 weeks, perhaps indicating initial evolution towards the cubic B_2_ phase. The return to higher Si, at 29 weeks, may signal a partial transition to the lower Ni T_7_ phase. Note these phase associations are speculative, since the extent of their composition fields are not known. For example, it is well established that Si and Mn are relatively interchangeable on their sub lattice in response to changes in the alloy bulk compositions^45,46^. Indeed, while TEM showed extra diffraction spots due to the presence of the precipitates, it was not possible to index them to specific phases, due to their still very small sizes and possible association with dislocations.

**Table 3:** Amount of Mn, Ni and Si in the matrix for the high Ni steel (CM6) from the AI and 425 °C annealed conditions.

*t*_*a*_	Average Matrix Composition (at.%)					
	Ni	Mn	Si	Mo	C	P
0	0.17	0.32	0.04	0.25	0.16	0.00
1	0.62	0.62	0.18	0.22	0.12	0.00
7	1.43	1.24	0.34	0.25	0.06	0.01
17	1.60	1.08	0.37	0.22	0.08	0.01
29	1.66	1.34	0.38	0.25	0.16	0.00

**Table 4:** Relative amount of Mn, Ni and Si in the precipitates and compared with known Mn-Ni-Si phases.

t_a_ (week)	Mn/Ni/Si	Closest Phase Mn/Ni/Si
0	0.35/0.53/0.12	Γ_2_ :≈ 34/47-52/14-20 XRD
1	0.36/0.54/0.10	Γ_2_ to B_2_ :≈ 41-47/50/3-9
7	0.38/0.54/0.08	Γ_2_ to B_2_ :≈ 41-47/50/3-9
17	0.39/0.54/0.07	Γ_2_ to B_2_ :≈ 41-47/50/3-9
29	0.41/0.42/0.17	Γ_2_ to T_7_ :≈ 50/33/17

Note phase name: Γ_2_ = T_6_ and G = T_3_.

It has been previously shown that tip-to-tip variability even in the same steel can be exploited to characterize the effects composition variations on precipitation^6,21^. The local composition also has a strong effect on the precipitate stability during PIA. For example, while MNSPs were still found in all the high Ni steel (CM6) tips in the AI, 1 week and 7 weeks t_a_ conditions, they were only found in tips containing more measured ≈1.5% Ni and 1.3% Mn bulk solutes (close to the nominal alloy composition) for longer *t*_*a*_. Due to the strong and very systematic effect of Ni and Mn, only tips that contained close to nominal alloy composition were included in plots of <*r*>, *f* and *N*. The full data for all runs taken is provided in the Supplemental Material (Tables S1 and S2) to more clearly demonstrate the tip-to-tip bulk composition variability and the corresponding impact on precipitate stability. It was also observed that small amounts of Mo and C at a ratio ≈1.25 (MoC to Mo_2_C), are co-segregated to the MNSPs following the 29 weeks anneal. Note, other studies have shown these elements are depleted in the MNSPs in the AI condition at lower temperature^8^. Finally, we again note that the TEM-EDS results are qualitatively consistent with the 29 weeks APT data.

### Cluster dynamics modeling

**Figure 8:**
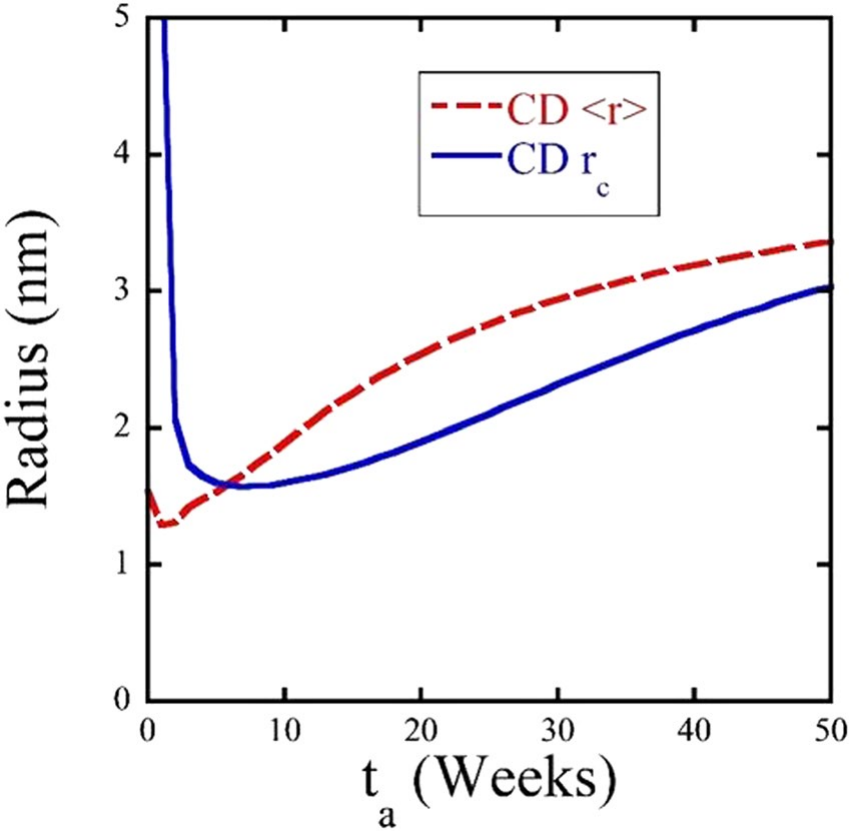
Cluster dynamics average precipitate radius (<*r*>) and calculated critical radius (*r*_*c*_) as a function of annealing time. Simulation conditions are the same as in Fig. 5.

Figure 8 illustrates the complex physics of precipitate annealing at T_a_ much higher than in their nearly fully decomposed formation condition at lower temperatures in terms of the CD model predictions of critical radius (*r*_*c*_) versus *t*_*a*_ compared to <*r*>. The critical radius is *r*_*c*_ = 2γ/*ΔG*_*v*_, where γ is the MNSP interface energy and ΔG_v_ is the volumetric dissolved minus precipitate free energy difference for the matrix composition at t_a_. Figure 6 shows that the <*r*> is initially far below *r*_*c*_ at 425 °C in the solute depleted matrix. However, the rapid re-solution of the Mn, Ni and Si, results in a corresponding rapid decrease in *r*_*c*_, with increasing t_a_, while <*r*> increases following an initial dip. In this case the CD model predicts that the <*r*> and *r*_*c*_ curves cross at ≈5 weeks. At this point *f* begins to increase, initially by growth and subsequently by coarsening with decreasing N. The CD model predicts that the cross over occurs at <*r*> = *r*_*c*_ ≈1.5 nm. In other cases the intersection could be delayed in t_a_ and at a larger <*r*> = *r*_*c*_, while in other cases there would be no intersection at all, leading to full dissolution. This delicate interaction between <*r*> and *r*_*c*_ leads to the high sensitivity of f(t_a_) shown in Fig. 7. Thus it is useful to try to estimate actual *r*_*c*_.

**Figure 9:**
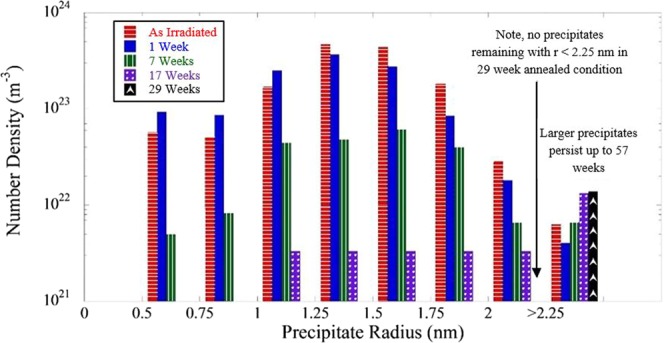
Size distribution of precipitates in the high Ni steel (CM6) for the AI and annealed conditions. Note that no precipitates with *r* < 1 nm were observed after 17 weeks of annealing and no precipitates with *r* < 2.25 nm were observed after 29 weeks of annealing.

The histogram plot Fig. 9 shows that the APT *N* continuously decreases with t_a_ at all MNSP sizes except for *r* > 2.25 nm. Note, these *N* and corresponding *f* are highly uncertain at the long annealing times, since at most, only a few precipitates are observed in a given APT tip. While precise precipitate size distributions cannot be established, the APT results show that the largest MNSP survive and persist and slightly increase in numbers at longer t_a_. Notably, no clusters with *r* < 2.25 nm were found in the 29 weeks condition. The largest MNSP in the AI condition was *r* = 2.3 nm, while the 3 precipitates found after the 29 weeks PIA all had *r* > 2.6 nm. The largest MNSP in the AI condition had ≈4500 solute atoms, the 3 clusters found after the 29 weeks PIA contained 6500, 7200 and 8100 solute atoms, respectively. Thus the MNSP with *r* > 2.25 nm are not only stable, but appear to be growing, supporting the hypothesis that they are equilibrium phases. This interpretation of the APT results is consistent and supported by the EDS observations described previously, which showed a much larger number of coarsened MNSPs persist after annealing for 57 weeks in areas with sufficient Ni and Mn. Based on these results it appears that as parameterized the CD model *r*_*c*_ ≈ 1.5 nm at the intersection with <*r*> underestimates the actual *r*_*c*_ which is closer to 2.3 nm. These results along with those in Fig. 8, suggest that at 425 °C the solvus boundary is larger than, for example, 1.5 at.% Ni and 1.0 at.% Mn and lower than 1.6 at.% Ni and 1.3 at.% Mn, since these compositions bracket the complete dissolution and overestimation of f, respectively.

The full dissolution in the medium Ni steel (LG) at 7 weeks demonstrates that 57 weeks t_a_ is far more than that needed to dissolve the MNSPs in the high Ni steel (CM6) if they are non-equilibrium solute clusters. The combination of APT results and CD model suggests that the significant reduction in the precipitate *N* is consistent with the nano-scale size of the MNSPs, which are predominantly below *r*_*c*_ at 425 °C in the initially solute depleted AI matrix. As the small MNSPs dissolve, the corresponding increase in the matrix solute concentration is sufficient to stabilize only the larger precipitates. The fact that MNSPs with *r* > 2.3 nm are stable in a matrix that is only slightly solute depleted (≈−0.11%) compared to the total solutes available in the AI condition, suggests that they are not induced by radiation, consistent with thermodynamic predictions, especially for much lower temperatures (see below). Thus, the overall CD predictions are qualitatively consistent with the observations, supporting the thermodynamic basis for the model and the interpretation of the PIA data. However, the CD model over predicts the number of stable precipitates that remain based on an independent (not fitted) γ and ΔG_v_, that underpredict *r*_*c*_ at 425 °C. Note, the PIA data could be used to fine-tune both precipitation and annealing models. However, such fitting is beyond the scope of this paper.

Finally, it is very important to return to the key question: *are the MNSPs stable, thermodynamic phases at a typical RPV service irradiation temperatures that are much lower than 425 °C?* This is clearly the case, since the CALPHAD ΔG_v_ is ≈4 times larger at 290 °C. Thus the corresponding r_c_ would be 0.54 nm, consistent, with the lower end of the observed MNSP size distribution in the AI condition.

## Conclusions

PIA was used to investigate the character of MNSPs in neutron irradiated RPV steels. Annealing at 425 °C for long times, resulted in complete precipitate dissolution in a medium Ni (0.69%) steel after only 7 weeks, *consistent with thermodynamic predictions*. In contrast, some MNSPs still remained at the longest annealing time of 57 weeks in the high Ni (1.6%) steel, again qualitatively consistent with the CD model predictions. APT showed that the local regions with the highest precipitate stability contained at least 1.6 at.% Ni and 1.4 at.% Mn, although even in these regions a significant reduction in *N* and *f* were observed. However, the MNSP N with *r* > 2.25 nm coarsened slightly, while all the smaller precipitates dissolved at long t_a_. This suggests that the critical radius r_c_ at 425 °C at the higher Ni and Mn contents is ≈2.3 nm. The number of Mn-Ni-Si atoms in the MNSPs found at 29 weeks averaged almost 50% higher than the largest cluster found in the AI condition, again indicating modest coarsening. Notably, the order of the t_a_ dependence of the increase in <*r*> and decrease in N is also consistent with classical diffusion controlled coarsening. Finally, TEM-EDS even more clearly showed stable precipitates in grains with sufficient Ni and Mn, even after annealing at 425 °C for 57 weeks, again strongly supporting the conclusion that MNSPs are a thermodynamically stable phase.

The <*r*>, *N* and *f* predicted by unfitted CD model is only qualitatively consistent with the experimental APT trends. While the model accurately predicts the increases in <*r*>, it overestimates the corresponding *N* and *f*. However, the model clearly reveals the basic PIA mechanism mediating the reduction in *N* and *f* is the large critical radius, *r*_*c*_, at 425 °C in the initially AI solute depleted matrix. The precipitates smaller than *r*_*c*_ dissolve and re-enrich the matrix. Hence, <*r*> increases with t_a_ while *r*_*c*_ decreases and after they intersect some of the remaining larger precipitates subsequently continue grow and coarsen even at the higher temperature of 425 °C. The CD model over predictions of *f* can be traced to its independent parameterization that predicts an *r*_*c*_ that is smaller than that observed.

The key issue that is being addressed in this work is the thermodynamic stability of the MNSPs at much lower irradiation temperatures. Since ΔG_v_ is of order 4 times larger, and *r*_*c*_ is 4 times smaller, at 290 °C compared to 425 °C, there can be no question that MNSPs a stable thermodynamics phase at such service relevant temperatures. Notably, these conclusions are consistent with both CALPHAD thermodynamic predictions and XRD measurements^21,27^.

Finally, we note that these results do not mean that solute (Mn-Ni-Si) segregation, including that driven by RIS, does not play a role in MNSP evolution at lower temperatures. Indeed they clearly do especially in the nucleation stage where these solutes segregate to small dislocation loops created in displacement cascades. Depending on the alloy composition, even if bulk MNSP phases (G and Γ_2_) are thermodynamically stable, slow homogeneous nucleation rates may greatly limit precipitation. There are many APT observations of heterogeneous nucleation on loops, line dislocations and grain boundaries, especially at lower alloy solute contents and or higher irradiation temperatures. Indeed, RIS is the likely cause of solute cluster formation, widely identified as a generic G-phase, in highly sub saturated alloys^6,40,47–49^.

## Supplementary information

Supplementary Material

## Data Availability

The datasets generated during and/or analyzed during the current study are not publicly available due to proprietary reasons but are available from the corresponding author on reasonable request. The full data for all CM6 runs analyzed during this study is provided in the supplemental material.
