# Supplementary material for: On the Elevated Temperature Thermal Stability of Nanoscale Mn-Ni-Si Precipitates Formed at Lower Temperature in Highly Irradiated Reactor Pressure Vessel Steels
Source: Sci Rep. 2019 Jul 3;9:9587. doi: 10.1038/s41598-019-45944-z (PMC6610118; doi:10.1038/s41598-019-45944-z)
Supplement: Supplementary file 1 — Supplementary Material [file 41598_2019_45944_MOESM1_ESM.pdf]

Supplementary Material for:

On the Elevated Temperature Thermal Stability of Nanoscale Mn-Ni-Si Precipitates Formed at Lower Temperature in Highly Irradiated Reactor Pressure Vessel Steels

N. Almirall[a], P. B. Wells[a,1], H. Ke[b,2], P. Edmondson[c], D. Morgan[b], T. Yamamoto[a], G. R. Odette[a]

[a)] Materials Department, University of California, Santa Barbara, CA 93106
[b)] Department of Materials Science and Engineering Department, University of Wisconsin, Madison, WI 53706
[c)] Materials Science and Technology Division, Oak Ridge National Laboratory, Oak Ridge, TN 37831
1) Current affiliation: Intel Corporation, Hillsboro, OR 97124
2) Current affiliation: Materials Science and Engineering Department, Ohio State University, Columbus, OH, 43210

1. PRECIPITATE VOLUME FRACTION VS MOLE FRACTION

The low evaporation potential of Mn-Ni-Si precipitates in an Fe matrix results in changes in local magnification and resulting in a focusing of matrix atoms into the precipitate region on the detector and is signaled by higher than physical atom densities in the reconstructed dataset [1, 2]. These artifacts also result in the distortions of the compositions, shape, and size of precipitates, and most specifically their apparent Fe content, which actually comes from the adjoining matrix and contributes to the higher than physical atom density in the precipitate itself [3, 4]. Using the number of solute atoms to define the cluster size minimizes these field evaporation distortions. For example, the MNSP mole fraction (f) was defined as the number of solute atoms in the clusters divided by the total number of atoms in the analyzed volume. Mole fraction (f) can be converted to volume fraction (f_v) if the density of the precipitate phase is known, but since there are two likely potential phases for the precipitates in the as-irradiated condition, and the MNSPs may possibly transform to a different phase under annealing, the mole fraction is given in this paper. Though a range of lattice constants for these phases are reporting in literature, using lattice constants of 1.1158 nm and 0.6687 for the G and Γ_2 phases [5], respectively, gives mole fractions are only ≈ 2% or ≈ 5% higher than for Fe.

2. ATOM PROBE TOMOGRAPHY DATA

Atom probe tomography (APT) data showed that there are large variations in solute content from region to region. These local solute contents directly influenced the precipitate stability, with higher solute content regions showing stable precipitates, while lower solute content regions showed completed dissolution. The tables below give the measured bulk, matrix and precipitate compositions for each

individual atom probe tip along with the measured precipitate values for those tips where precipitates were observed.

Table S1. APT bulk and matrix compositions for all measured CM6 tips. Note that tips without any precipitation have dashes in the matrix composition as they were the same as the bulk composition.

Run Number	Condition	Bulk Composition (at.%)						Matrix Composition (at.%)					
		Fe	Ni	Mn	Si	C	Mo	Fe	Ni	Mn	Si	C	Mo
2179	AI	96.04	1.62	1.34	0.38	0.23	0.25	98.96	0.15	0.28	0.04	0.21	0.26
2180	AI	95.63	1.76	1.42	0.44	0.31	0.22	98.66	0.20	0.30	0.06	0.28	0.22
2185	AI	95.70	1.90	1.37	0.43	0.22	0.31	98.82	0.27	0.30	0.05	0.21	0.28
2186	AI	96.47	1.42	1.19	0.32	0.26	0.22	98.98	0.16	0.25	0.04	0.23	0.22
2493	AI	95.82	1.74	1.43	0.39	0.32	0.24	98.98	0.16	0.29	0.04	0.28	0.22
2495	AI	95.99	1.71	1.37	0.40	0.20	0.26	98.96	0.20	0.31	0.04	0.17	0.23
2496	AI	95.90	1.73	1.52	0.38	0.17	0.24	99.03	0.15	0.32	0.04	0.16	0.24
2497	AI	96.15	1.57	1.40	0.38	0.16	0.25	99.00	0.15	0.34	0.04	0.14	0.25
2498	AI	96.02	1.69	1.50	0.40	0.08	0.27	99.06	0.18	0.35	0.04	0.07	0.27
3246	425°C/1 Wk	96.41	1.59	1.15	0.38	0.22	0.20	97.11	1.21	0.93	0.31	0.20	0.20
3252	425°C/1 Wk	96.34	1.49	1.30	0.37	0.21	0.25	98.72	0.26	0.42	0.09	0.20	0.25
3270	425°C/1 Wk	96.78	1.37	1.16	0.33	0.08	0.23	98.54	0.45	0.54	0.15	0.06	0.23
3272	425°C/1 Wk	96.68	1.38	1.19	0.34	0.09	0.24	98.53	0.41	0.52	0.14	0.08	0.23
3273	425°C/1 Wk	96.71	1.39	1.10	0.35	0.14	0.22	98.01	0.69	0.66	0.22	0.12	0.22
3274	425°C/1 Wk	96.76	1.38	1.17	0.33	0.07	0.23	98.80	0.32	0.43	0.11	0.07	0.23
3742	425°C/7 Wks	96.16	1.64	1.41	0.36	0.13	0.26	96.52	1.49	1.28	0.34	0.11	0.23
3745	425°C/7 Wks	96.30	1.63	1.37	0.37	0.07	0.25	96.62	1.45	1.25	0.34	0.06	0.25
3766	425°C/7 Wks	96.29	1.64	1.37	0.36	0.05	0.25	96.72	1.40	1.22	0.33	0.05	0.25
3920	425°C/17 Wks	96.36	1.63	1.38	0.34	0.05	0.23	96.46	1.58	1.34	0.33	0.04	0.22
3923	425°C/17 Wks	96.96	1.49	1.01	0.34	0.02	0.17	-	-	-	-	-	-
3929	425°C/17 Wks	96.67	1.71	0.72	0.45	0.18	0.26	-	-	-	-	-	-
3930	425°C/17 Wks	96.38	1.66	1.27	0.36	0.07	0.21	-	-	-	-	-	-
4275	425°C/29 Wks	96.54	1.53	1.27	0.36	0.09	0.20	96.68	1.48	1.21	0.34	0.08	0.20
4328	425°C/29 Wks	96.16	1.66	1.34	0.38	0.16	0.25	96.35	1.58	1.25	0.36	0.13	0.28
4276	425°C/29 Wks	96.77	1.44	1.30	0.33	0.06	0.08	-	-	-	-	-	-
4277	425°C/29 Wks	97.03	1.33	1.10	0.30	0.08	0.15	-	-	-	-	-	-

**Trace amounts of P and Cr also present, but not listed.*

Table S2. APT Precipitate composition and <r>, N and f for all measured CM6 tips. Note that any tips with dashes did not have any measured precipitation.

Run Number	Condition	Precipitate Composition (at.%)						Precipitate <r>, N, f		
		Fe	Ni	Mn	Si	C	Mo	<r> (nm)	N (10^{23} m^{-3})	f (%)
2179	AI	60.58	20.33	13.95	4.50	0.24	0.23	1.53	19.02	2.77
2180	AI	56.66	22.49	15.06	5.26	0.25	0.12	1.43	22.99	2.92
2185	AI	61.21	21.28	11.87	4.97	0.23	0.26	1.43	21.17	2.82
2186	AI	57.96	21.72	14.68	4.96	0.29	0.25	1.50	16.56	2.31
2493	AI	58.02	21.80	14.46	4.88	0.44	0.27	1.54	20.09	2.86
2495	AI	60.53	20.59	13.55	4.70	0.24	0.25	1.50	20.30	2.81
2496	AI	57.35	21.87	15.58	4.68	0.20	0.21	1.61	17.44	2.97
2497	AI	59.76	20.67	14.27	4.81	0.17	0.20	1.51	19.46	2.68
2498	AI	57.52	21.62	15.50	4.94	0.11	0.22	1.54	19.50	2.89
3246	425°C/1 Wk	57.88	22.86	13.70	4.65	0.33	0.26	1.26	7.05	0.60
3252	425°C/1 Wk	64.20	18.86	12.47	3.82	0.20	0.27	1.53	14.25	2.12
3270	425°C/1 Wk	61.72	20.28	13.65	3.75	0.13	0.28	1.46	11.51	1.50
3272	425°C/1 Wk	62.09	20.04	13.53	3.76	0.10	0.26	1.43	12.96	1.59
3273	425°C/1 Wk	61.51	20.57	13.47	3.72	0.15	0.28	1.34	12.05	1.19
3274	425°C/1 Wk	62.02	20.03	13.63	3.81	0.07	0.26	1.45	14.41	1.79
3742	425°C/7 Wks	52.66	22.66	19.71	3.33	0.41	0.89	2.06	1.04	0.31
3745	425°C/7 Wks	55.45	23.52	16.76	3.64	0.16	0.32	1.56	1.98	0.33
3766	425°C/7 Wks	59.72	21.76	15.02	3.07	0.05	0.28	1.57	2.70	0.43
3920	425°C/17 Wks	55.84	23.21	16.59	3.12	0.38	0.70	2.12	0.30	0.12
3923	425°C/17 Wks	-	-	-	-	-	-	-	-	-
3929	425°C/17 Wks	-	-	-	-	-	-	-	-	-
3930	425°C/17 Wks	-	-	-	-	-	-	-	-	-
4275	425°C/29 Wks	35.72	22.72	22.85	9.04	3.25	5.82	2.78	0.10	0.09
4328	425°C/29 Wks	40.93	21.48	20.53	8.65	3.04	4.72	2.64	0.25	0.14
4276	425°C/29 Wks	-	-	-	-	-	-	-	-	-
4277	425°C/29 Wks	-	-	-	-	-	-	-	-	-

**Trace amounts of P and Cr also present, but not listed.*

3. EDS LINE SCAN

While the TALOS provided very high resolution elemental maps showing MNSPs remaining in the high Ni steel (CM6) at an annealing time of 57 weeks, the quantitative analysis was not consistent with APT as all maps had measured Mn contents of > 3.5 at.%, which is significantly higher than any region seen in APT (or expected based on the known composition of the alloy, CM6). While future work will focus

on better calibrating the TALOS for more quantitative measurement, in the short term, the FEI Titan at UCSB was used to perform a line scan and determine the local solute content of the grain containing very large precipitates and compare this with the neighboring grains, which did not contain and precipitates.

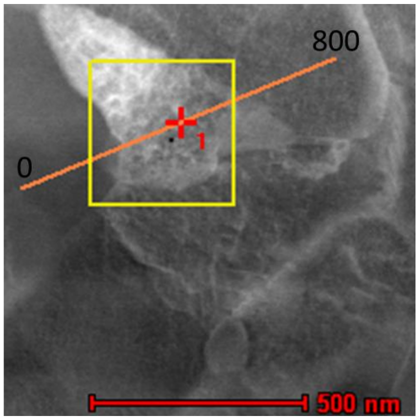

Figure S1. Location of line scan taken in the FEI Titan at UCSB where the region centered in the box contained a high density of large MNSPs as measured by the TALOS at ORNL (Figure 5, bottom in the text).

4. REFERENCES

1. Miller M.K. and Russell K.F. Embrittlement of RPV steels: An atom probe tomography perspective *J. Nucl. Mater.* 2007,**371**(1–3),145–160.

2. Marquis E.A. and Hyde J.M. Applications of atom-probe tomography to the characterisation of solute behaviours *Mater. Sci. Eng. R Reports* 2010,**69**(4–5),37–62.

3. Cunningham NJ. "Study of the Structure, Composition, and Stability of Y-Ti-O nm-Scale Features" [PhD Thesis]. University of California, Santa Barbara, 2012.

4. Vurpillot F., Bostel A., Blavette D. Trajectory overlaps and local magnification in three-dimensional atom probe", *Appl. Phys. Lett.* 2000,**76**(21),3127–3129.

5. Gupta K.P. The Mn-Ni-Si (Manganese-Nickel-Silicon) System *J. Phase Equiilibria Diffus.* 2006,**27**(5),529–534.
